# Emotion-specific regulation components differentially predict profiles of adolescent psychosocial dysfunction

**DOI:** 10.1038/s41598-026-46321-3

**Published:** 2026-03-31

**Authors:** Ahmad Asgarizadeh, Maryam Tahan, Farzaneh Ebrahimi, Narges Mazaheri, David G. Weissman, Katherine Dixon-Gordon

**Affiliations:** 1 Faculty of Education and Psychology, Shahid Beheshti University, Shahid Shahriari Square, Daneshjou Boulevard, Shahid Chamran Highway, Tehran, Iran; 2 Hillan Research Center, Tehran, Iran; 3 Faculty of Psychology and Education, University of Tehran, Tehran, Iran; 4 Faculty of Social Welfare and Health Sciences, Islamic Azad University, Tehran Medical Sciences Branch, Tehran, Iran; 5 Department of Psychology, California State University, Dominguez Hills, Carson, CA USA; 6 Department of Psychological and Brain Sciences, University of Massachusetts Amherst, Amherst, MA USA

**Keywords:** Emotion regulation, Emotion dysregulation, Adolescent psychopathology, LASSO regression, Cluster analysis, Diseases, Health care, Psychology, Psychology, Risk factors

## Abstract

Emotion regulation (ER) difficulties are a transdiagnostic risk factor for adolescent psychopathology, yet research is hampered by high intercorrelations among ER components. This study employed a data-driven approach to identify distinct profiles of adolescent dysfunction and select a parsimonious set of stable emotion-specific regulatory predictors. A sample of 795 Iranian adolescents (68.6% female; M_age_ = 16.06) completed measures of emotion-specific ER across sadness, fear, and anger (CERSA), psychosocial adjustment (SDQ), pathological traits (PID-5-BF), and disability (WHODAS-12). Person-centered cluster analysis identified dysfunction profiles, and ordinal LASSO penalized regression selected stable ER predictors of cluster membership, with both steps embedded within a full-pipeline bootstrap. Three stable clusters emerged, reflecting a severity gradient: low-, moderate-, and high-dysfunction. This solution was independently replicated via latent profile analysis sensitivity analysis (84.4% agreement, weighted κ = 0.86). LASSO regression with full-pipeline bootstrap inference retained eight stable predictors from 21 candidates, with seven threshold-specific effects supported by unconditional 95% confidence intervals excluding zero. General dysregulation across all three emotions was the strongest maladaptive predictor. In addition to general deficits, specific strategies showed context-dependent effects: Rumination-Anger was associated with greater dysfunction, whereas Reappraisal-Anger and Support Seeking-Sadness predicted lower dysfunction severity. Some associations were specific to particular severity transitions (low to moderate or moderate to high dysfunction) rather than uniform across the dysfunction spectrum. Our findings indicate that general dysregulation may represent transdiagnostic vulnerability, whereas specific strategies show emotion–context dependency, supporting functionalist theories that adaptiveness depends on matching regulatory strategies to an emotion’s function.

**Supplementary Information:**

The online version contains supplementary material available at 10.1038/s41598-026-46321-3.

## Introduction

Adolescence is a developmental period of significant transformation in the psychological processes governing emotion regulation (ER)^1,2^. This stage is characterized by a convergence of neurobiological and social changes, including the continued maturation of the prefrontal cortex, which lags behind the more affectively reactive limbic system^3^, and a heightened sensitivity to the evolving social landscape of peer evaluation^4^. This combination creates a period of both unique vulnerability and opportunity for psychosocial adjustment.

Within this developmental landscape, difficulties in managing emotional experiences emerge as well-established transdiagnostic risk factors for a wide range of psychological disorders^5,6^. Whereas emotion regulation refers to efforts to influence the quality, timing, intensity, or expression of one’s emotions^7^, emotion dysregulation refers to tendencies to experience or express emotions in a manner that interferes with appropriate goal-directed behaviors^8^. Importantly, contemporary process models conceptualize emotion dysregulation as a distinct class of regulatory failure rather than as a mere restatement of symptom severity^9^. Longitudinal evidence in adolescents supports this distinction by demonstrating the temporal precedence of dysregulation; latent dysregulation factors predict subsequent increases in anxiety, aggression, and eating pathology even after controlling for baseline symptoms, whereas baseline symptoms fail to predict later changes in dysregulation^10^. While these findings establish dysregulation as a primary vulnerability factor, the relationship can evolve into a transactional, bidirectional process once symptoms are established^11^. Within this cycle, initial regulatory failures elevate the risk for psychopathology, which may in turn further erode an adolescent’s capacity for effective ER. How adolescents learn to manage the normative rise in negative emotions within this complex, self-perpetuating system is therefore a critical factor in the onset and maintenance of psychopathology^12^.

Characterizing these self-perpetuating pathways requires moving beyond a global view of emotion, as the impact of dysregulation is rarely uniform across different emotional states. Consequently, research has shifted from viewing ER as a unitary ability to focusing on how the regulation of specific negative emotions is differentially associated with psychological outcomes^1,13^. However, this emotion-specific approach has revealed a complex and often overlapping picture. For example, while poor anger regulation is a known predictor of aggressive behavior, and sadness dysregulation is strongly linked to depression^14^, certain patterns of anger dysregulation are also associated with internalizing symptoms such as depression and anxiety^13^. Furthermore, the trajectory for fear/anxiety is particularly complex, as some models propose that anxiety functions not as a primary emotion to be regulated, but as a secondary signal of other rising emotions, which it then interferes with and dysregulates^15^. Taken together, these counterintuitive patterns reinforce the argument for a sophisticated, emotion-specific analytic framework that can identify both the maladaptive and the adaptive correlates of discrete regulatory processes.

This complexity presents a significant analytical challenge in studying the association between ER and psychopathology/maladjustment. ER is a multifaceted construct^7^, and simultaneously assessing the regulation of multiple emotions across various abilities/strategies confronts researchers with a large set of highly correlated potential predictors. Recent person-centered approaches have begun to address this issue by identifying distinct profiles of adolescents based on their patterns of use of specific ER strategies^13^. Such strategies include antecedent-focused approaches such as cognitive reappraisal, which involves reframing an emotional situation to alter its impact, and response-focused strategies such as expressive suppression, which involves inhibiting outward emotional displays. Another key process is rumination, which is characterized by a repetitive and passive focus on the causes and consequences of negative affect^7^.

While this work effectively shows that different ER profiles exist and are related to psychopathology, a critical question remains: which specific ER components are the most potent predictors of adolescent dysfunction? Traditional analytical approaches, such as standard multiple regression, struggle to disentangle the relative importance of these intercorrelated variables. To address this issue, advanced statistical methods offer distinct advantages. Penalized regression techniques, particularly the least absolute shrinkage and selection operator (LASSO), are well-suited for this purpose. LASSO performs simultaneous variable selection and regularization by applying a penalty that shrinks the coefficients of less influential predictors (in some cases, exactly to zero). This data-driven approach is designed to identify a parsimonious set of core predictors from a larger pool, thereby enhancing model interpretability and reducing the risk of overfitting^16^. The goal is not only to find predictors but also to distinguish the most influential ER components from those with negligible predictive value in the presence of others. By systematically identifying which specific regulatory processes are most critical, such methods can clarify the complex interplay between emotion-specific regulation and adolescent psychopathology.

This study addresses the challenge of identifying the most salient ER components for predicting adolescent dysfunction from a large set of intercorrelated variables. We employed a data-driven, two-stage approach to address this issue. First, a person-centered cluster analysis was used to establish distinct profiles of adolescent psychopathology and maladjustment. Second, we applied LASSO penalized regression to 21 ER components, which encompass seven regulatory processes (Distraction, Reappraisal, Expressive Suppression, Support Seeking, Rumination, Control, and Dysregulation) assessed across three emotional scenarios that induce sadness, fear, and anger. The primary objective was to select a parsimonious and stable set of the most critical predictors of cluster membership, thereby specifying the ER components most strongly associated with adolescent psychopathology/maladjustment. Given the intercorrelated nature of these ER facets and the discovery-oriented purpose of cluster analysis and LASSO regression, this study was designed to be data-driven and exploratory; therefore, we did not specify a priori hypotheses.

## Methods

### Participants

The participants in this study were 795 seventh- to twelfth-grader adolescents recruited from selected schools in Tehran (68.6% female; M_age_ = 16.06, SD = 1.23, age range = 12–18 years). The detailed sociodemographic characteristics of the sample are presented in Table 1. The responses were initially screened for completeness, with any submission missing over 10% of the items being discarded. To ensure data integrity, several quality control procedures were implemented to detect and remove careless responses. These included the use of three embedded instructed items (flagging responses with one or more incorrect answers), the identification of excessive identical responses (longstring analysis, where more than half of the items received the same answer), and the calculation of person‒total correlations (excluding negative values)^17^.

**Table 1:** Sociodemographic characteristics of participants (*N* = 795).

Characteristics		Frequency	Percentage
Sex	Female	545	68.6
	Male	250	31.4
Grade	7th	36	4.5
	8th	10	1.3
	9th	32	4
	10th	322	40.5
	11th	196	24.7
	12th	195	24.5
	Prefer not to say	3	0.4
Birth order	First-born	418	52.6
	Second-born	265	33.3
	Third- and later-born	106	13.3
	Prefer not to say	6	0.8
Number of siblings	0	87	10.9
	1	413	51.9
	2+	253	31.8
	Prefer not to say	42	5.3
Diagnostic status	Diagnosed	43	5.4
	Non-diagnosed	692	87
	Prefer not to say	60	7.5

From an initial pool of 1169 collected responses, 374 were excluded because of quality issues. Specifically, 48 responses were removed because of excessive missing data, 129 because of failed instructed-item checks, 1 for exhibiting a longstring pattern, and 196 because of negative person‒total correlations. While a 32% exclusion rate may seem high, elevated careless responses are common in adolescent samples, with prevalence estimates ranging from 14.17% to 37.45%, depending on the stringency of the exclusion criteria^18^. Moreover, our use of multiple, complementary indices increases detection accuracy and strengthens the validity of the screened dataset^17^. Consistent with the abovementioned developmental trend, our comparison between retained and excluded participants also revealed a pattern: no significant differences were observed regarding sex, birth order, number of siblings, or diagnostic status (*ps* = 0.09‒0.66), but excluded participants were younger and in lower grades (*ps* < 0.001). Because this age-related attrition could theoretically introduce selection bias, we conducted a sensitivity analysis by re-running our clustering procedures on the full, un-screened dataset. As detailed in Sect.  3.2, both our primary clustering approach and a formal model-based latent profile analysis applied to this inclusive sample corroborated the extraction of three profiles, demonstrating that the structural findings are not artifacts of the data cleaning process.

This study, conducted in accordance with the ethical guidelines of the Declaration of Helsinki, was approved by Tehran’s Education Administration of District 2, Region 20, and the Raftar Cognitive Neuroscience Research Center Ethics Committee. Informed consent from both parents and adolescents was obtained for participation; failure to provide consent from either party resulted in exclusion from the study. Notably, a subset of this dataset was previously used in a separate publication with distinct research objectives^19^.

### Procedures

Data collection occurred between April and December 2024. The research team utilized a convenience sampling approach to select schools in Tehran. Prior to initiating data collection, formal authorization was obtained from the administration of each participating school. School personnel facilitated communication with parents, informing them of the study’s aims and enabling them to provide consent. Before providing their own informed consent, the students were acquainted with the study’s objectives and were assured of the confidentiality of their responses.

Data collection was conducted within the participating schools during a standard class period. The entire battery of instruments was administered in a single session lasting approximately 60 min to ensure consistency and minimize cohort effects. These sessions were supervised by a trained member of the research team, with the classroom teacher also present. The participants were instructed to complete the questionnaires independently to maintain response confidentiality and were able to proceed at their own pace. The supervising researcher was available throughout the session to clarify any questions or concerns that arose.

### Measures

A battery of instruments assessing ER components, psychosocial adjustment, pathological traits, and general disability was administered (Table 2; see Supplementary Information, Sect.  1 for full descriptions). Three instructed response items were embedded throughout the battery, each directing participants to select a specific response option (e.g., “Please select the ‘Totally like me’ option”) to identify careless responses. Additionally, details on sex, age, current grade level, birth order, number of siblings, and self-reported diagnostic status (i.e., whether they were currently diagnosed and receiving treatment from a child psychiatrist/psychologist, or not diagnosed) were collected. The present investigation utilizes a dataset derived from a more comprehensive research project, the primary aims of which were entirely distinct from the objectives pursued herein.

**Table 2:** An overview of the study measures.

Measure	Validity/reliability evidence	Intended construct	Number of Items	Subscales (number of items)	Used score / scale
Contextualized Emotion Regulation Survey for Adolescents – Sadness scale (CERSA-Sadness)^32^	Good to excellent internal consistency (ω generally ranging from 0.78 to 0.92 across subscales and studies). Validity supported by a confirmed seven-factor structure, good construct and convergent validity (e.g., relationships with other ER measures), and strong criterion-related validity (e.g., associations with life satisfaction, psychopathology, and functional impairment); measurement invariance across demographic groups such as sex and birth order^32^^a^.	Sadness regulation	27	Distraction (3), Reappraisal (4), Expressive Suppression (4), Support Seeking (4), Rumination (4), Control (4), and Dysregulation (4)	Seven subscale scores / Likert-type (1–7)
Contextualized Emotion Regulation Survey for Adolescents – Fear scale (CERSA-Fear)^32^		Fear regulation	27	Distraction (3), Reappraisal (4), Expressive Suppression (4), Support Seeking (4), Rumination (4), Control (4), and Dysregulation (4)	Seven subscale scores / Likert-type (1–7)
Contextualized Emotion Regulation Survey for Adolescents – Anger scale (CERSA-Anger)^32^		Anger regulation	27	Distraction (3), Reappraisal (4), Expressive Suppression (4), Support Seeking (4), Rumination (4), Control (4), and Dysregulation (4)	Seven subscale scores / Likert-type (1–7)
Self-report Strengths and Difficulties Questionnaire (SDQ)^54^	Good total difficulties reliability (α often > 0.80), subscale-level 0.68–0.77. Extensive construct and criterion-related validity (e.g., correlates with CBCL, clinical diagnoses); sensitive to change^54,55^.	Psychosocial adjustment	25	Internalizing problems (10), Externalizing problems (10), and Prosociality (5)	Three subscale scores / Likert-type (0–2)
Personality Inventory for DSM-5 – Brief Form (PID-5-BF)^56^	α generally 0.65–0.85 (can be lower, e.g., Detachment α = 0.59 in adolescents); Adequate 2-month test-retest reliability (ICCs 0.78–0.97). Five-factor structure is supported; good convergent and discriminant validity^57,58^.	Pathological traits	25	Negative Affectivity (5), Detachment (5), Antagonism (5), Disinhibition (5), and Psychoticism (5)	Five subscale scores / Likert-type (0–3)
12-item self-administered World Health Organization disability assessment schedule 2.0 (WHODAS-12)^59^	High internal consistency (α often ≥ 0.80 ). Good construct and convergent validity with other disability measures; invariance across youth with and without physical/mental conditions; differentiates by employment status and medical comorbidities^59,60^.	Disability	12	-	Total score / Likert-type (1–5)

DSM = Diagnostic and Statistical Manual of Mental Disorders, CBCL = Child Behavior Checklist.

^a^To confirm that the CERSA scenarios elicited their intended target emotions within our sample, we conducted three separate repeated-measures ANOVAs. The results confirmed that each scenario primarily induced the correct emotion (see Supplementary Information, Sect.  2, Table S1).

### Data analysis

#### Cluster analysis

Analyses were conducted via SPSS v. 27 and RStudio v. 2024.09. All psychopathology measures (SDQ subscales: Prosociality, Internalizing, Externalizing; PID-5-BF domains: Negative Affectivity, Detachment, Antagonism, Disinhibition, Psychoticism; and WHODAS-12 total disability score) and all 21 ER components from the CERSA (seven regulatory processes across three emotions) were z-standardized prior to analysis to place them on a common scale. To identify subgroups of adolescent dysfunction, we then employed a two-stage clustering procedure using nine standardized input variables: the three SDQ subscales, five PID-5-BF trait domains, and the WHODAS-12 total score. In the first stage, hierarchical clustering was carried out via Ward’s linkage and squared Euclidean distances, yielding preliminary cluster centroids. In the second stage, these centroids served as initial seeds for a nonhierarchical k-means algorithm, which iteratively refined group membership to minimize within-cluster variance^20^.

The optimal cluster number was initially determined through (1) dendrogram inspection, which involves identifying a long branch without further merging (the preceding merge indicates the last meaningful cluster join), and (2) agglomeration schedule inspection, which involves locating the step with a pronounced jump in coefficients (again, the stage just before this jump marks the final merge). Because such criteria can involve subjective interpretation, we also applied the NbClust package v. 3.0.1^21^, which computes multiple indices and selects the cluster solution supported by the majority of the indices. Finally, we used the fpc package v. 2.2–13^22^ to test the stability of the identified clusters, with a mean Jaccard similarity value > 0.75 indicating stability^23^.

#### LASSO regression and full-pipeline bootstrap inference

To identify the ER components most strongly associated with adolescent dysfunction clusters while addressing post-selection inference, we implemented a full-pipeline approach: ordinal LASSO regression was used for variable selection, followed by unpenalized partial proportional-odds estimation on the selected subset, with both steps embedded within a stratified bootstrap (B = 10000, preserving cluster proportions). Since the Brant test yielded a significant result (χ^2^_(21)_ = 43.44, *p* < .001), cluster membership was modeled via a nonproportional odds cumulative-logit model throughout, permitting each predictor to have a unique effect across the two outcome thresholds.

The ordinal LASSO was fitted with all 21 CERSA subscales as predictors (seven regulatory processes × three emotions) via the ordinalNet package v. 2.1.2^24^. Ordinal LASSO regression is a penalized regression technique suitable for predicting an ordinal outcome variable while simultaneously performing variable selection and regularization. Tuning of the LASSO penalty (λ) employed 10-fold cross-validation^16^. This process evaluates model performance across different values of λ to identify the values that provide the best balance between model fit and parsimony. Two candidate values were estimated: (1) the λ_min_ value corresponds to the λ that resulted in the minimum average cross-validated log-likelihood, indicating the model with the best predictive performance on the training data; and (2) the λ_1se_ value represents the largest λ within one standard error of the minimum log-likelihood. This more conservative value often leads to a simpler, more parsimonious model that may generalize better to new data. Our primary inferences are based on λ_1se_, which is consistent with our aim of identifying a parsimonious set of key predictors rather than optimizing predictive accuracy^16,25^.

Importantly, in LASSO regression, coefficients that are shrunk to exactly zero signify that the corresponding predictor was not selected by the model at the given level of penalty, suggesting that it has a negligible unique contribution to predicting cluster membership in the presence of the other selected variables. The magnitude of the nonzero coefficients can be interpreted as reflecting the relative importance of each predictor in distinguishing between the clusters (however, it is important to note that LASSO coefficients are biased towards zero due to the shrinkage penalty and should not be interpreted as unbiased estimates of effect sizes^16^. For each predictor, the sum of absolute coefficients across all thresholds and the number of thresholds at which the coefficient was exactly zero in the initial full-data LASSO model are reported in Table 3 for ranking purposes only. Since our goal was variable selection (i.e., identifying a parsimonious set of key ER components) rather than optimizing prediction, we did not assess traditional predictive-performance indices.

**Table 3:** Ordinal LASSO regression predicting cluster membership and bootstrapped selection frequencies.

Variables	Ordinal LASSO		Bootstrapping		
	Σ|β| (λ_1se_)^a^	# Zero thresholds^b^	Selection frequency^c^	T1 selection frequency^d^	T2 selection frequency^e^
Dysregulation-anger	0.706	0	1.000	0.994	1.000
Dysregulation-fear	0.491	0	0.999	0.992	0.968
Dysregulation-sadness	0.466	0	1.000	0.998	0.952
Rumination-anger	0.359	0	0.997	0.983	0.844
Expressive suppression-sadness	0.293	0	0.993	0.812	0.965
Reappraisal-anger	0.225	1	0.990	0.261	0.986
Support seeking-sadness	0.220	1	0.991	0.983	0.427
Rumination-fear	0.210	0	0.972	0.773	0.891
Reappraisal-sadness	0.134	1	0.885	0.120	0.871
Expressive suppression-fear	0.118	1	0.890	0.868	0.159
Support seeking-fear	0.107	1	0.847	0.244	0.787
Reappraisal-fear	0.106	0	0.894	0.574	0.739
Support seeking-anger	0.100	0	0.906	0.805	0.452
Control-fear	0.048	1	0.737	0.12	0.689
Expressive suppression-anger	0.040	1	0.795	0.731	0.207
Distraction-sadness	0.038	0	0.813	0.586	0.556
Rumination-sadness	0.000	2	0.432	0.075	0.381
Control-sadness	0.000	2	0.509	0.315	0.267
Distraction-fear	0.000	2	0.514	0.244	0.337
Distraction-anger	0.000	2	0.545	0.459	0.170
Control-anger	0.000	2	0.374	0.257	0.148

^a^Sum of absolute coefficients across the two thresholds from the ordinal LASSO model at λ_1se_. LASSO penalized coefficients (∑|β|) are reported here for ranking purposes only and are subject to bias; these values should not be interpreted as unbiased effect-size estimates. Formal inference is conducted via the full-pipeline bootstrap procedure.

^b^Number of thresholds (out of two) for which the coefficient was shrunk exactly to zero at λ_1se_.

^c^Proportion of full-pipeline resamples (B = 10000) in which that predictor had a non-zero coefficient (at least for one threshold).

^d^Proportion of resamples with nonzero coefficient at threshold 1.

^e^Proportion of resamples with nonzero coefficient at threshold 2.

In the bootstrap (conducted to assess selection stability^26^, the LASSO was refit at the fixed λ₁ₛₑ in each resample, and an unpenalized partial proportional-odds model (VGAM package v. 1.1–13^27^) was then fit on that same resample using only the resampled-selected predictors. For each of the 21 predictors, the threshold-specific coefficient from this model was recorded if the predictor was selected, and set to zero otherwise. This procedure yields an unconditional bootstrap distribution for every threshold-specific coefficient (one that absorbs the full uncertainty of both variable selection and post-selection estimation) without conditioning on a fixed set of predictors. Unconditional 95% bootstrap confidence intervals (2.5th − 97.5th percentiles) served as the primary inferential criterion: an effect is considered supported when its interval excludes zero. The overall selection frequency (the proportion of resamples in which the LASSO assigned a nonzero coefficient to at least one threshold) was retained as a stability criterion; subscales selected in > 95% of resamples were classified as stable predictors.

## Results

### Descriptive analyses

Only 0.61% of the item responses were missing; cases with any missing scale data were excluded listwise when computing scale means and z-scores, an amount too small to affect our findings materially. All LASSO regression assumptions were tested and confirmed (Supplementary Information, Sect.  3, Tables S2 and S3). Supplementary Table S4 presents descriptive statistics and internal consistency coefficients for the study variables.

### Cluster analysis

Hierarchical methods consistently pointed to triadic grouping in our data. Inspection of the dendrogram alongside the agglomeration schedule revealed that the optimal cutoff occurred three instances before the final merger, yielding three clusters (see Supplementary Figs. S2 and S3). However, the results from NbClust were not decisive: of the total 24 indices, 11 suggested a two-cluster solution, whereas 10 supported a three-cluster solution. Importantly, when we applied this same procedure to the full, un-screened dataset as a sensitivity analysis, the support shifted slightly in favor of the three-cluster solution (12 indices supported three clusters, whereas 10 supported two clusters). While the two-cluster solution separated a high-pathology/low-prosociality group from a low-pathology/high-prosociality group, we selected the three-cluster solution by seeking an optimal trade-off across multiple cluster-validity criteria. First, our hierarchical clustering (dendrogram inspection and agglomeration-coefficient jump) revealed three clusters, with NbClust serving as supplementary rather than determinative guidance. This prioritization is defensible because no single validity index is universally superior, and different indices quantify different desirable clustering characteristics^28^. Second, this three-cluster solution accords with precedent in adolescent psychopathology research: data-driven analyses of both community and clinical samples commonly recover not only low- and high-risk groups but also intermediate or “mild-risk” profiles^29,30^.

**Fig. 1:**
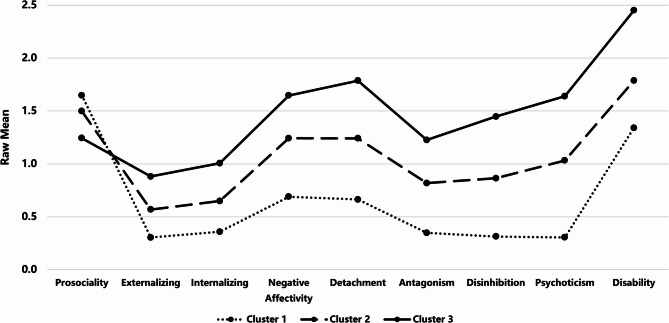
Raw mean scores of dysfunction indicators for each cluster. Cluster 1 = *Low-dysfunction*, Cluster 2 = *Moderate-dysfunction*, Cluster 3 = *High-dysfunction*.

Consequently, we applied k-means with *k* = 3, seeding centroids from the hierarchical cluster centers. All the clusters presented acceptable mean Jaccard values (0.91–0.95), confirming the stability of the three-cluster solution when the k-means algorithm was used. As expected given the clustering algorithm, all input variables showed large separation across clusters: Prosociality (η^2^ = 0.149), Internalizing (η^2^ = 0.438), Externalizing (η^2^ = 0.461), Negative Affectivity (η^2^ = 0.326), Detachment (η^2^ = 0.387), Antagonism (η^2^ = 0.310), Disinhibition (η^2^ = 0.503), Psychoticism (η^2^ = 0.496), and Disability (η^2^ = 0.472). Moreover, a chi-square test indicated a significant association between cluster membership and diagnostic status, χ²_(2)_ = 11.46, *p* = .003, Cramer’s *V* = 0.12, further supporting the external validity of the clustering solution. Cluster 1 (*N* = 223) presented the highest level of prosociality and the lowest degree of dysfunction, Cluster 2 (*N* = 322) presented an intermediate level, and Cluster 3 (*N* = 250) presented the most severe dysfunction. Accordingly, we labeled these clusters as *low-dysfunction*, *moderate-dysfunction*, and *high-dysfunction* (Fig. 1).

A model-based sensitivity analysis using latent profile analysis replicated this three-profile solution, with 84.4% agreement between the two methods (weighted κ = 0.86; see Supplementary Information, Sect.  4).

### LASSO regression

The ordinal LASSO regression model, with λ_1se_ = 0.01986 (see Supplementary Information, Figure S1 for the cross-validation plot), identified 16 of the 21 CERSA subscales as predictors of adolescent dysfunction cluster membership (Table 3). Among these, Dysregulation across all three emotions exhibited the largest overall effect magnitudes. These three subscales, along with six others (Rumination-Anger, Expressive-Suppression-Sadness, Rumination-Fear, Reappraisal-Fear, Support Seeking-Anger, and Distraction-Sadness), demonstrated effects across both ordinal thresholds (nonzero at both thresholds). The remaining seven selected subscales were associated with only a single threshold. The coefficients of the five CERSA subscales were shrunk to zero by the λ_1se_ ​model.

Bootstrap analysis was used to assess selection stability at the fixed λ_1se_​. Of the 16 CERSA subscales initially retained by the λ₁ₛₑ model, eight met the predefined stability criterion in the full-pipeline bootstrap (B = 10000): Dysregulation-Anger, Dysregulation-Sadness, Dysregulation-Fear, Rumination-Anger, Support Seeking-Sadness, Expressive Suppression-Sadness, Reappraisal-Anger, and Rumination-Fear; the remaining eight subscales failed to exceed the 95% threshold (see Table 3). The retained subscales were carried forward for bootstrap inference.

### Full-pipeline bootstrap inference

A full-pipeline bootstrap inference for all eight stable predictors is presented in Table 4 (see Table S5 for the results of all the predictors). Unconditional 95% CIs excluded zero for seven of the sixteen threshold-specific effects. All three Dysregulation subscales decreased the odds of falling into the low-dysfunction cluster at the first threshold (low vs. moderate/high dysfunction): Dysregulation-Sadness (OR = 0.720, 95% CI [0.520, 0.866]), Dysregulation-Fear (OR = 0.782, 95% CI [0.544, 0.964]), and Dysregulation-Anger (OR = 0.744, 95% CI [0.532, 0.924]). Dysregulation-Anger additionally showed a robust effect at the second threshold (OR = 0.637, 95% CI [0.496, 0.795]); the corresponding CIs for Dysregulation-Sadness and Dysregulation-Fear at the second threshold included zero. Rumination-Anger was associated with greater dysfunction at the first threshold (OR = 0.751, 95% CI [0.595, 0.946]). On the other hand, Support Seeking-Sadness increased the odds of remaining in the low-dysfunction cluster at the first threshold (OR = 1.383, 95% CI [1.082, 1.938]), and Reappraisal-Anger increased the odds of remaining in the low- or moderate-dysfunction clusters at the second threshold (OR = 1.350, 95% CI [1.073, 1.740]). The remaining threshold-specific effects had unconditional CIs that included zero, indicating that post-selection inference does not support these effects at the threshold level.

**Table 4:** Full-pipeline bootstrap inference for the eight stable emotion regulation components predicting cluster membership (B = 10000).

Predictor	Threshold	Estimate^a^	95% Bootstrap CI^b^	OR^c^	95% OR CI
Dysregulation-anger	logit [*P* ≤ 1]	–0.400	**[− 0.631**,** − 0.079]**	0.744	[0.532, 0.924]
	logit [*P* ≤ 2]	–0.480	**[− 0.701**,** − 0.229]**	0.637	[0.496, 0.795]
Dysregulation-fear	logit [*P* ≤ 1]	–0.362	**[− 0.610**,** − 0.040]**	0.782	[0.544, 0.964]
	logit [*P* ≤ 2]	–0.231	[− 0.429, 0.000]	0.825	[0.651, 1.000]
Dysregulation-sadness	logit [*P* ≤ 1]	–0.480	**[− 0.654**,** − 0.144]**	0.720	[0.520, 0.866]
	logit [*P* ≤ 2]	–0.298	[− 0.454, 0.000]	0.798	[0.635, 1.000]
Rumination-anger	logit [*P* ≤ 1]	–0.313	**[− 0.519**,** − 0.056]**	0.751	[0.595, 0.946]
	logit [*P* ≤ 2]	–0.217	[− 0.426, 0.000]	0.862	[0.653, 1.000]
Expressive suppression-sadness	logit [*P* ≤ 1]	–0.095	[− 0.331, 0.014]	0.909	[0.718, 1.014]
	logit [*P* ≤ 2]	–0.286	[− 0.480, 0.000]	0.772	[0.619, 1.000]
Reappraisal-anger	logit [*P* ≤ 1]	0.080	[0.000, 0.259]	1.000	[1.000, 1.296]
	logit [*P* ≤ 2]	0.313	**[0.070**,** 0.554]**	1.350	[1.073, 1.740]
Support seeking-sadness	logit [*P* ≤ 1]	0.441	**[0.079**,** 0.662]**	1.383	[1.082, 1.938]
	logit [*P* ≤ 2]	0.090	[0.000, 0.324]	1.000	[1.000, 1.383]
Rumination-fear	logit [*P* ≤ 1]	–0.174	[− 0.383, 0.000]	0.886	[0.682, 1.000]
	logit [*P* ≤ 2]	–0.209	[− 0.433, 0.000]	0.812	[0.649, 1.000]

OR = odds ratio of cluster membership at the given threshold (exp of unconditional bootstrap median). CIs excluding zero are bold-faced.

^a^Point estimates are unpenalized coefficients from the full-data model, representing the best available effect size estimates given the observed variable selection; because the CIs are derived from a zero-inflated unconditional distribution rather than the full-data estimate, they are asymmetric around the point estimates. This is expected and by design, not an error

^b^Bootstrap CIs are unconditional (mass-at-zero for non-selected resamples included); they account for full-pipeline selection uncertainty by incorporating both the probability of a predictor being selected and the uncertainty of its post-selection estimate.

^c^Because the cumulative logit model predicts the probability of being in a lower severity category, an OR < 1 indicates that higher predictor scores are associated with a higher risk of severe dysfunction, whereas an OR > 1 indicates increased odds of remaining in a lower-dysfunction cluster.

### Sensitivity analysis

To evaluate the robustness of our findings, we conducted a sensitivity analysis using the two-cluster solution. The complete analytical pipeline (including LASSO variable selection and full-pipeline bootstrap inference) was applied to these two clusters. The detailed results are provided in Supplementary Tables S6 and S7. In this model, Dysregulation (across Anger, Fear, and Sadness), Reappraisal-Anger, and Expressive Suppression-Sadness remained highly stable (selection frequencies > 97.7%) and statistically meaningful (95% CIs excluded zero). Notably, Expressive Suppression-Sadness reached inferential support in the two-cluster model despite its threshold-specific CIs, which included zero in the three-cluster model. In contrast, Rumination-Anger and Support Seeking-Sadness, which met stability thresholds and reached inferential support in the three-cluster model, fell below the 95% stability threshold in the two-cluster framework.

## Discussion

This study first identified three distinct and stable profiles of adolescent functioning in a community sample, which we labeled low-, moderate-, and high-dysfunction. The clear severity gradient across these profiles supports a dimensional, transdiagnostic view of psychopathology rather than discrete diagnostic categories, aligning with concepts such as the general factor of psychopathology (p-factor) that captures shared liability across mental health domains^31^. The primary goal was then to determine which specific ER processes most strongly differentiated these groups.

As captured by the CERSA items, general “Dysregulation” reflects a state in which emotions overflow and get the better of the individual, leading to actions that go beyond thoughts and a subjective sense of being unable to control reactions even when they are recognized as inappropriate^32^. Our findings indicated that general Dysregulation (across anger, sadness, and fear) was the strongest and most consistent predictor of membership in the higher-dysfunction profiles. The importance of general Dysregulation was further corroborated by a sensitivity analysis using a two-cluster solution, where dysregulation across all three emotional contexts remained a highly stable predictor of cluster membership. Beyond this overarching component, a parsimonious and stable set of specific ER strategies also predicted profile membership. The findings highlighted the maladaptive role of Rumination-Anger (first threshold), alongside the protective associations of Reappraisal-Anger (second threshold) and Support Seeking-Sadness (first threshold). Expressive Suppression-Sadness and Rumination-Fear also exhibited high selection stability but their threshold-specific CIs included zero, warranting cautious, exploratory interpretation.

The finding that general Dysregulation served as the strongest predictor aligns with evidence that it is a central transdiagnostic factor in adolescent psychopathology. For example, longitudinal data from a two-year follow-up study demonstrated that emotion dysregulation prospectively predicted increases in a general psychopathology factor, acting as a key mechanism linking childhood maltreatment to psychopathology over time^33^. In a large nonclinical adolescent sample, Iannattone, et al.^34^ further reported that dysregulation uniquely predicts both internalizing and externalizing symptoms, accounting for substantial variance beyond mediators such as boredom and problematic social media use. Converging neural and clinical evidence also reinforces this view, showing that dysregulation is associated with altered neurobiological markers and functions as a central mechanism in dimensional models of psychopathology^35^.

It is important to interpret the predictive value of the general Dysregulation subscales with appropriate caution. Measurement concerns have been raised regarding potential conceptual overlap between dysregulation items and psychopathology symptoms, as self-report measures may inadvertently index distress-laden experiences alongside regulatory failures^36,37^. However, despite these measurement challenges, substantial evidence supports dysregulation as a valid transdiagnostic construct. For example, bifactor analyses reveal that a general dysregulation factor prospectively predicts treatment outcomes even after accounting for item-level criterion contamination^38^. Furthermore, neurophysiological evidence has demonstrated that trait dysregulation measures exhibit significant statistical covariation with objective neural markers of emotional responding, such as late positive potential. As these associations persist independent of specific diagnostic categories, they provide evidence that self-report dysregulation scales capture the underlying neurobiological signatures of affective processing rather than merely reflecting concurrent symptom distress^39^. Several aspects of the present measurement strategy further mitigate this concern. The CERSA items are strictly anchored in specific emotional contexts and emphasize the process of control loss (e.g., emotions “overflowing”) rather than enumerating discrete psychiatric symptoms (e.g., sleep disturbance, rule-breaking behaviors, callousness, and general disability) which are captured by our outcome measures, the SDQ, PID-5-BF, and WHODAS-12. Therefore, while acknowledging inherent conceptual overlap, evidence suggests that the strong association observed here reflects a transdiagnostic liability for dysfunction rather than a measurement artifact.

In addition to the important role of general dysregulation, our findings suggest emotion-specific patterns in the adaptiveness of ER strategies, in line with functionalist theories of emotion. However, given that emotional responses were assessed via scenario-based self-reports rather than direct indices of emotional responding, these patterns should be interpreted as context-dependent associations rather than definitive evidence of emotion-specificity. In the context of anger, cognitive reappraisal was associated with a lower risk of severe dysfunction, a finding supported by meta-analytic evidence linking it to lower anger intensity^40^. Crucially, this dovetails with findings that anger impairs perspective-taking due to elevated arousal and that promoting perspective-taking helps mitigate anger’s interpersonal consequences^41^. Moreover, it aligns with evidence identifying cognitive reappraisal as a potent mechanism for down-regulating anger intensity, underscoring its relevance as a target for therapeutic intervention^40^. Conversely, rumination in anger contexts was identified as one of the most stable correlates of greater dysfunction, aligning with data that repetitive negative thinking sustains emotional arousal and contributes to both internalizing and externalizing difficulties^42^.

In the context of sadness, support-seeking was particularly adaptive, which is consistent with the functionalist notion that sadness elicits social connection and with systematic studies linking it to lower depression and anxiety^43^. On the other hand, Expressive Suppression-Sadness showed high overall selection stability but its threshold-specific bootstrap CIs included zero at both thresholds, yielding only tentative, inconclusive evidence for a maladaptive association. Theoretically, suppression may interfere with sadness’s adaptive function of eliciting social support^44^: when adolescents hide their sad expressions, they may disrupt a natural social communication system and rely on a strategy that is ineffective for down-regulating sadness itself. Given its selection stability, and the convergent evidence from the two-cluster sensitivity analysis (in which its effect reached inferential support under the simpler binary contrast) this association warrants investigation in larger confirmatory samples.

In the context of fear, rumination showed high bootstrap selection stability but its threshold-specific CIs included zero at both thresholds, providing only tentative support for a maladaptive association. However, its stability is consistent with longitudinal findings that perseverative negative thinking undermines recovery from threats and contributes to ongoing anxiety^45^. From a functionalist perspective, fear serves crucial adaptive functions centered on threat detection and protective action, operating along a threat imminence continuum that evokes specific behaviors optimized for each threat level^46^. Fear promotes rapid physiological mobilization and action-oriented responses essential for survival, functions conserved across 700 million years of evolution^47^. Rumination in fear contexts is therefore particularly maladaptive because it directly opposes these adaptive functions through repetitive, abstract negative thinking that potentially hinders concrete protective action^48^. Moreover, rumination maintains physiological arousal long after threats have passed through what Brosschot, et al.^49^ termed “perseverative cognition,” which prolongs stress-related activation even in the absence of actual stressors. This sustained arousal may impede the natural recovery that should follow adaptive fear responses, potentially contributing to chronic anxiety^50^; a process that is theoretically plausible but not inferentially supported by the present bootstrap results.

Beyond the main effects, the full-pipeline bootstrap inference revealed two threshold-specific patterns that illuminate how certain ER strategies may differentially relate to transitions across the psychopathology spectrum. First, anger rumination was associated with dysfunction only at the first threshold (low- vs. moderate/high-dysfunction), suggesting that it may be particularly salient during initial transitions into psychopathology. This aligns with evidence that rumination is a transdiagnostic risk factor influencing illness trajectories from their earliest stages^51^ and with research in young adults supporting anger rumination as a transdiagnostic correlate of psychopathology. Conversely, cognitive reappraisal of anger was linked to dysfunction only at the more severe, second threshold (low/moderate- vs. high-dysfunction). This pattern is consistent with evidence that reappraisal’s benefits are most pronounced under high-stress conditions, where it may be crucial for mitigating symptom escalation^52^, and with findings that it can moderate the relationship between risk factors and severe outcomes such as non-suicidal self-injury^53^.

The necessity of this granular, three-cluster approach was indicated through our sensitivity analysis. When the sample collapsed into a two-cluster framework, Rumination-Anger and Support Seeking-Sadness, both of which reached inferential support in the three-cluster model, fell below the 95% stability threshold. This suggests that while general dysregulation is a stable predictor, the three-cluster model is uniquely sensitive to the strategy-specific effects that characterize the transitions between moderate and severe levels of adolescent dysfunction. However, as our design is cross-sectional, prospective replication is needed on two fronts: to establish whether the supported threshold-specific patterns reflect genuine causal or temporal pathways, and to determine whether the tentative associations observed for Expressive Suppression-Sadness and Rumination-Fear are reliable.

A central strength of the present study is its methodological and analytic innovation. By combining person-centered cluster analysis with LASSO penalized regression, we identified naturally occurring profiles of adolescent adjustment and then isolated a parsimonious, stable set of the most critical emotion regulation components that differentiated these profiles. This dual-stage, data-driven approach addresses a long-standing challenge in the field by effectively managing the high intercorrelations among regulatory components. Nevertheless, these findings should be interpreted in light of several limitations. The cross-sectional design restricts causal inference, as the relationship between dysregulation and psychopathology is likely transactional, meaning that our findings represent associations rather than predictive pathways. Our reliance on self-report measures may also inflate these associations due to shared method variance. Furthermore, the emotion coverage of the CERSA is limited to sadness, fear, and anger; self-conscious emotions relevant to adolescent psychopathology, such as shame and guilt, were not assessed, partly because no validated adolescent measures of their context-specific regulation currently exist. Additionally, while the CERSA scenarios successfully induced their target emotions, the resulting emotional states were not entirely pure, which introduces some overlap in the regulatory context being assessed. Finally, the sample was drawn exclusively from Iranian adolescents and was predominantly female (68.6%), which may limit the generalizability of our findings across different cultural and gender contexts. Future research should address these issues through longitudinal, multi-method designs to clarify causal pathways and capture the dynamic nature of regulation. Cross-cultural replication in more gender-balanced samples is essential to test whether the emotion-specific adaptiveness of strategies can be generalized across diverse sociocultural contexts. Finally, applying these methods in clinical samples would also strengthen their translational value, helping to refine and validate specific intervention targets for evidence-based treatments.

## Conclusion

Employing a novel data-driven approach that combines person-centered clustering with LASSO regression, our study clarifies a central tension in emotion regulation research. We found that while general dysregulation may represent a transdiagnostic vulnerability marker, the adaptiveness of specific regulatory strategies is highly context-dependent. Specifically, the analysis revealed context-dependent patterns: rumination was maladaptive in the context of anger, whereas reappraisal and support seeking predicted lower dysfunction severity in the contexts of anger and sadness, respectively. Rumination-Fear and Expressive Suppression-Sadness showed high selection stability but did not reach inferential support. These results reconcile competing theoretical frameworks by showing that effective regulation requires both a foundational regulatory capacity and emotional agility to deploy strategies that align with an emotion’s evolved function. Ultimately, if replicated in longitudinal and clinical samples, these findings may point toward a dual-focus intervention approach that simultaneously targets general regulatory deficits while building the context-specific skill of matching strategies to emotions.

## Supplementary Information

Below is the link to the electronic supplementary material.

Supplementary Material 1

## Data Availability

The dataset collected and analyzed during the current study is not publicly available as it jeopardizes the privacy and consent of research participants, but is available from the corresponding author upon reasonable request.
