# Supplementary material for: Emotion-specific regulation components differentially predict profiles of adolescent psychosocial dysfunction
Source: Sci Rep. 2026 Mar 31;16:15591. doi: 10.1038/s41598-026-46321-3 (PMC13187445; doi:10.1038/s41598-026-46321-3)
Supplement: Supplementary file 1 — Supplementary Material 1 [file 41598_2026_46321_MOESM1_ESM.docx]

**Supplementary Information: Emotion-Specific Regulation and Adolescent Psychosocial Dysfunction: A Person-Centered LASSO Approach**

**Ahmad Asgarizadeh, Maryam Tahan, Farzaneh Ebrahimi, Narges Mazaheri, David G. Weissman, Katherine Dixon-Gordon**

**Section 1. Description of Measures**

To evaluate ER facets in adolescents, we administered the Contextualized Emotion Regulation Survey for Adolescents (CERSA; Fombouchet et al., 2023). This instrument uniquely presents participants with emotion-specific scenarios before assessing their regulatory responses, rather than measuring ER globally. Three distinct scales target regulation of sadness (prompted by a scenario about a close friend’s departure from school), fear (elicited through a scenario involving forgotten speech content), and anger (provoked by a scenario describing a friend’s betrayal of confidential information). Although each scale contains the same 27 items measuring five strategies (Reappraisal, Expressive Suppression, Distraction, Support Seeking, and Rumination) and two abilities (Controlling Experience and Dysregulation), participants rate them separately following each emotional context using a seven-point response format (1 = *Not at all like me* to 7 = *Totally like me*). Research has confirmed robust psychometric properties for the CERSA scales, including strong associations with conventional ER assessments and life satisfaction measures (Fombouchet et al., 2023).

Psychosocial adjustment was measured using the Strengths and Difficulties Questionnaire (SDQ; Goodman, 1997), a widely-utilized screening tool for youth populations. This 25-item self-report measure employs a three-point response format (*Not true*, *Somewhat true*, *Certainly true*) to capture both difficulties and prosocial functioning in children and adolescents. Although Goodman's original framework proposed five distinct factors, empirical evidence has increasingly favored a three-factor model comprising Externalizing, Internalizing, and Prosocial dimensions, especially when applied to community samples with low psychopathology risk (Goodman et al., 2010). This streamlined structural configuration has received empirical support, demonstrating adequate internal consistency and construct validity (Ruchkin et al., 2008).

Pathological personality traits aligned with the AMPD framework (American Psychiatric Association, 2013) were assessed using the Personality Inventory for DSM-5 – Brief Form (PID5BF; Krueger et al., 2013). This instrument captures five maladaptive trait domains: negative affectivity, detachment, antagonism, disinhibition, and psychoticism. Respondents rate items using a 4-point response format ranging from very false or often false to very true or often true, with higher subscale scores indicating greater trait manifestation. The instrument's five-factor structure has received substantial empirical support across multiple investigations, which have confirmed its convergent and divergent validity, reliability coefficients, and gender-based measurement invariance (Anderson et al., 2018; Gomez et al., 2022). Psychometric evaluation of the Persian adaptation has similarly yielded satisfactory properties (Elhami Athar & Ebrahimi, 2022).

General disability was assessed through the World Health Organization Disability Assessment Schedule 2.0 (WHODAS; Üstün et al., 2010), specifically utilizing its 12-item abbreviated form (WHODAS-12). This measure captures dysfunction over the previous month across six fundamental life domains: mobility, cognition, life activities, self-care, social participation, and interpersonal relationships. Participants indicate their level of difficulty on a five-point response scale from 1 (*None*) to 5 (*Extreme or cannot do*). The WHODAS-12 has demonstrated robust psychometric characteristics when administered to adolescent community samples (Kimber et al., 2015; Tompke et al., 2020). For analytical purposes in this investigation, we calculated a composite total score across all items.

**Section 2. Comparing Emotion Ratings for Each CERSA Scenario**

To confirm that the CERSA scenarios elicited their intended target emotions within our sample, we conducted three separate repeated-measures ANOVAs. The results confirmed that each scenario primarily induced the correct emotion (Supplementary Table S1). For the sadness-inducing scenario, adolescents reported significantly higher levels of sadness than fear or anger. Similarly, the fear-inducing scenario elicited significantly more fear than sadness or anger, neither of which differed from the other. Finally, the anger-inducing scenario elicited significantly more anger than both sadness and fear.

**Section 3. Assumptions of the LASSO regression**

**Sample size requirements.** In accordance with classical events-per-variable (EPV) guidelines, we calculated EPV at each cumulative split of our three-category outcome. For the first split (Membership > 1), there are 572 events (clusters 2 + 3), yielding EPV ≈ 572 / 21 = 27.2; for the second split (Membership > 2), there are 250 events, yielding EPV ≈ 250 / 21 = 11.9—both above the traditional benchmarks. While the frequently cited “EPV ≥ 10” rule originates from early simulation studies (Peduzzi et al., 1996), later reviews and methodological texts recommend more conservative thresholds, typically 10–15 EPV, depending on context (Harrell, 2015; Heinze & Dunkler, 2017; Steyerberg, 2019). Though no formal EPV standard exists for ordinal regression, applying binary logistic EPV criteria to each cumulative split is a common and practical approach.

High-dimensional sparsity theory for the LASSO further requires that the number of nonzero coefficients *s* satisfy:

*s* ≪ *n* / log(*p*),

where *n* = 795 and *p* = 21, so *n* / log(*p*) ≈ 795 / log(21) ≈ 261. Provided *s* is substantially smaller than 261, theoretical guarantees for consistency and support recovery are preserved (Bühlmann & van de Geer, 2011). These conditions are comfortably met in our case, as the dimensionality is modest and the sample size-to-variable ratio (*n/p* ≈ 38) is well above typical thresholds for stable penalized estimation. Together, these benchmarks confirm that our sample size (*N* = 795) is more than adequate for fitting an ordinal LASSO model with 21 predictors.

**Multicollinearity assessment.** We conducted an ordinary least-squares regression that included all 21 z-scored CERSA subscales and examined variance-inflation factors (VIFs) and tolerances. All VIFs ranged from 1.75 to 2.31 (tolerance = 0.43–0.57), well below the commonly used cutoff of 5, indicating no problematic multicollinearity (Supplementary Table S2).

**Linearity in logit.** This assumption was assessed via the Box–Tidwell test for each predictor at each cumulative split, with a conservative α = 0.01 to reduce false positives due to multiple comparisons. All predictors showed no evidence of non-linearity at this level, so they were retained as linear terms in our penalized model (Supplementary Table S3).

**Multivariate outliers.** Using a stringent Mahalanobis cutoff (*p* < .001; Tabachnick & Fidell, 2019), we flagged 21 of 795 observations (2.6 %) as multivariate outliers on the z-standardized CERSA subscales. Our careless responding indices flagged none of these cases, suggesting they reflect genuine yet extreme ER profiles. Accordingly, they were retained in the analyses.

**Proportional odds.** As reported in the manuscript, a significant omnibus Brant test suggested non-parallel terms (χ^2^_(21)_ = 43.44, *p* < .001).

**Section 4. Sensitivity analysis using latent profile analysis**

To evaluate the robustness of the derived clusters, a Latent Profile Analysis (LPA) was conducted as a model-based sensitivity analysis using the tidyLPA package (v. 2.0.2; Rosenberg et al., 2018). Two model parameterizations were estimated for 1–5 profiles: Model 1 (equal variances, zero covariances) and Model 2 (varying variances, zero covariances), using the same nine standardized indicators as the primary clustering (Scrucca et al., 2016). The optimal number of profiles was determined by inspecting the BIC elbow and the Bootstrapped Likelihood Ratio Test (BLRT) in accordance with contemporary best practice guidelines for finite mixture modeling (Nylund-Gibson et al., 2023).

The LPA sensitivity analysis supported a three-profile solution across both model parameterizations. The BIC showed a pronounced elbow at three profiles for both Model 1 (ΔBIC = −299 from 2 to 3 profiles, vs. −80 from 3 to 4) and Model 2 (ΔBIC = −484 vs. −45), with the BLRT significant at each step (*p* =.0099). Model 2 (varying variances, zero covariances) was selected as optimal based on superior BIC (18,037 vs. 18,405) and higher classification precision (Entropy = 0.85) relative to Model 1. Profile sizes were well-balanced (38.0%, 43.0%, and 19.0%), with no splinter classes. Cross-tabulation of LPA maximum-posterior-probability assignments with the original clustering assignments revealed 84.4% agreement, with substantial to near-perfect concordance (weighted κ = 0.86, 95% CI [0.84, 0.89]; Landis & Koch, 1977). This high degree of convergence between the heuristic and model-based approaches supports the structural validity of the three-cluster solution.

**References**

American Psychiatric Association. (2013). *Diagnostic and Statistical Manual of Mental Disorders* (5th ed.). American Psychiatric Publishing.

Anderson, J. L., Sellbom, M., & Salekin, R. T. (2018). Utility of the Personality Inventory for DSM-5-Brief Form (PID-5-BF) in the Measurement of Maladaptive Personality and Psychopathology. *Assessment*, *25*(5), 596-607. <https://doi.org/10.1177/1073191116676889>

Bühlmann, P., & van de Geer, S. (2011). *Statistics for High-Dimensional Data: Methods, Theory and Applications*. Springer Berlin, Heidelberg. <https://doi.org/10.1007/978-3-642-20192-9>

Elhami Athar, M., & Ebrahimi, A. (2022). Validation of the Personality Inventory for DSM-5–Brief Form (PID-5-BF) with Iranian University Students and Clinical Samples: Factor Structure, Measurement Invariance, and Convergent, Discriminant, and Known-Groups Validity. *Journal of Personality Assessment*, 1-11. <https://doi.org/10.1080/00223891.2022.2152347>

Fombouchet, Y., Lannegrand, L., & Lucenet, J. (2023). The Contextualized Emotion Regulation Survey for Adolescents (CERSA): How does emotion regulation vary according to context? *British Journal of Developmental Psychology*, *41*(3), 306-323. <https://doi.org/10.1111/bjdp.12450>

Gomez, R., Watson, S., Brown, T., & Stavropoulos, V. (2022). Personality inventory for DSM–5-Brief Form (PID-5-BF): Measurement invariance across men and women. *Personality Disorders: Theory, Research, and Treatment*, No Pagination Specified-No Pagination Specified. <https://doi.org/10.1037/per0000569>

Goodman, A., Lamping, D. L., & Ploubidis, G. B. (2010). When to use broader internalising and externalising subscales instead of the hypothesised five subscales on the Strengths and Difficulties Questionnaire (SDQ): data from British parents, teachers and children. *J Abnorm Child Psychol*, *38*(8), 1179-1191. <https://doi.org/10.1007/s10802-010-9434-x>

Goodman, R. (1997). The Strengths and Difficulties Questionnaire: a research note. *J Child Psychol Psychiatry*, *38*(5), 581-586. <https://doi.org/10.1111/j.1469-7610.1997.tb01545.x>

Harrell, F. E. J. (2015). *Regression Modeling Strategies: With Applications to Linear Models, Logistic and Ordinal Regression, and Survival Analysis* (2nd ed.). Springer Cham. <https://doi.org/10.1007/978-3-319-19425-7>

Heinze, G., & Dunkler, D. (2017). Five myths about variable selection. *Transpl Int*, *30*(1), 6-10. <https://doi.org/10.1111/tri.12895>

Kimber, M., Rehm, J., & Ferro, M. A. (2015). Measurement Invariance of the WHODAS 2.0 in a Population-Based Sample of Youth. *PLOS ONE*, *10*(11), e0142385. <https://doi.org/10.1371/journal.pone.0142385>

Krueger, R. F., Derringer, J., Markon, K. E., Watson, D., & Skodol, A. E. (2013). The personality inventory for DSM-5—brief form (PID-5-BF)—adult. *Washington, DC: American Psychiatric Association*.

Landis, J. R., & Koch, G. G. (1977). The measurement of observer agreement for categorical data. *Biometrics*, *33*(1), 159-174.

Nylund-Gibson, K., Garber, A. C., Carter, D. B., Chan, M., Arch, D. A. N., Simon, O., Whaling, K., Tartt, E., & Lawrie, S. I. (2023). Ten frequently asked questions about latent transition analysis. *Psychol Methods*, *28*(2), 284-300. <https://doi.org/10.1037/met0000486>

Peduzzi, P., Concato, J., Kemper, E., Holford, T. R., & Feinstein, A. R. (1996). A simulation study of the number of events per variable in logistic regression analysis. *Journal of Clinical Epidemiology*, *49*(12), 1373-1379. <https://doi.org/10.1016/S0895-4356(96)00236-3>

Rosenberg, J. M., Beymer, P. N., Anderson, D. J., van Lissa, C. J., & Schmidt, J. A. (2018). tidyLPA: An R Package to Easily Carry Out Latent Profile Analysis (LPA) Using Open-Source or Commercial Software. *Journal of Open Source Software*, *3*(30), 978. <https://doi.org/10.21105/joss.00978>

Ruchkin, V., Jones, S., Vermeiren, R., & Schwab-Stone, M. (2008). The Strengths and Difficulties Questionnaire: the self-report version in American urban and suburban youth. *Psychol Assess*, *20*(2), 175-182. <https://doi.org/10.1037/1040-3590.20.2.175>

Scrucca, L., Fop, M., Murphy, T. B., & Raftery, A. E. (2016). mclust 5: Clustering, Classification and Density Estimation Using Gaussian Finite Mixture Models. *R j*, *8*(1), 289-317.

Steyerberg, E. W. (2019). *Clinical Prediction Models: A Practical Approach to Development, Validation, and Updating*. Springer Cham. <https://doi.org/10.1007/978-3-030-16399-0>

Tabachnick, B., & Fidell, L. (2019). *Using Multivariate Statistics* (7th ed.). Pearson Education.

Tompke, B. K., Tang, J., Oltean, II, Buchan, M. C., Reaume, S. V., & Ferro, M. A. (2020). Measurement Invariance of the WHODAS 2.0 Across Youth With and Without Physical or Mental Conditions. *Assessment*, *27*(7), 1490-1501. <https://doi.org/10.1177/1073191118816435>

Üstün, T. B., Kostanjsek, N., Chatterji, S., & Rehm, J. (2010). *Measuring health and disability: Manual for WHO disability assessment schedule WHODAS 2.0*. World Health Organization.

*Supplementary Table S1*

Results of Repeated Measures ANOVAs Comparing Emotion Ratings for Each CERSA Scenario

| **CERSA Scenario** | **Emotion Rating** | **M (SD)** | **Omnibus F-test** | **Partial η^2^** | **Post-Hoc Comparisons** |
| --- | --- | --- | --- | --- | --- |
| Sadness  (Best friend moving away) | Sadness | 4.13 (1.75) | F(1.94, 1492.22) = 708.49, p < .001 | .479 | Sadness > Fear, p < .001  Sadness > Anger, p < .001  Fear vs. Anger, p = .056 |
|  | Fear | 2.14 (1.55) |  |  |  |
|  | Anger | 2.03 (1.50) |  |  |  |
| Fear  (Forgetting a presentation) | Fear | 4.58 (1.96) | F(1.96, 1537.60) = 101.46, p < .001 | .114 | Fear > Sadness, p < .001  Fear > Anger, p < .001  Sadness vs. Anger, p = .430 |
|  | Sadness | 3.67 (1.99) |  |  |  |
|  | Anger | 3.62 (2.16) |  |  |  |
| Anger  (Friend reveals a secret) | Anger | 5.61 (1.76) | F(1.97, 1543.69) = 729.70, p < .001 | .483 | Anger > Sadness, p < .001  Anger > Fear, p < .001  Sadness > Fear, p < .001 |
|  | Sadness | 4.62 (2.01) |  |  |  |
|  | Fear | 2.64 (1.93) |  |  |  |
| ***Note.*** M = Mean; SD = Standard Deviation; Partial η² = Partial Eta Squared. Degrees of freedom were corrected using the Greenhouse-Geisser estimate due to violations of the sphericity assumption. Post-hoc pairwise comparisons were conducted with Fisher’s LSD method. | | | | | |

*Supplementary Table S2*

Collinearity diagnostics for z-standardized CERSA subscales

| **Variables** | **Tolerance** | **VIF** |
| --- | --- | --- |
| Distraction-Sadness | .531 | 1.884 |
| Reappraisal-Sadness | .561 | 1.781 |
| Expressive Suppression-Sadness | .538 | 1.859 |
| Support Seeking-Sadness | .455 | 2.197 |
| Rumination-Sadness | .505 | 1.981 |
| Control-Sadness | .570 | 1.754 |
| Dysregulation-Sadness | .543 | 1.843 |
| Distraction-Fear | .511 | 1.957 |
| Reappraisal-Fear | .471 | 2.125 |
| Expressive Suppression-Fear | .546 | 1.830 |
| Support Seeking-Fear | .465 | 2.150 |
| Rumination-Fear | .495 | 2.019 |
| Control-Fear | .476 | 2.100 |
| Dysregulation-Fear | .481 | 2.081 |
| Distraction-Anger | .433 | 2.307 |
| Reappraisal-Anger | .438 | 2.283 |
| Expressive Suppression-Anger | .504 | 1.984 |
| Support Seeking-Anger | .457 | 2.189 |
| Rumination-Anger | .464 | 2.154 |
| Control-Anger | .468 | 2.136 |
| Dysregulation-Anger | .514 | 1.947 |

*Supplementary Table S3*

Linearity in the Logit: Box–Tidwell Test for Non-linearity across cumulative splits

| **Predictor** | **Split** | ***p*‑value** |
| --- | --- | --- |
| Distraction‑Sadness | > 1 | .2273 |
| Reappraisal‑Sadness | > 1 | .6399 |
| Expressive Suppression-Sadness | > 1 | .3983 |
| Support Seeking‑Sadness | > 1 | .4972 |
| Rumination‑Sadness | > 1 | .6621 |
| Control‑Sadness | > 1 | .1708 |
| Dysregulation‑Sadness | > 1 | .9215 |
| Distraction‑Fear | > 1 | .1423 |
| Reappraisal‑Fear | > 1 | .3501 |
| Expressive Suppression‑Fear | > 1 | .2115 |
| Support Seeking‑Fear | > 1 | .8140 |
| Rumination‑Fear | > 1 | .3026 |
| Control‑Fear | > 1 | .1632 |
| Dysregulation‑Fear | > 1 | .5106 |
| Distraction‑Anger | > 1 | .2642 |
| Reappraisal‑Anger | > 1 | .6039 |
| Expressive Suppression‑Anger | > 1 | .2368 |
| Support Seeking‑Anger | > 1 | .4793 |
| Rumination‑Anger | > 1 | .4977 |
| Control‑Anger | > 1 | .6116 |
| Dysregulation‑Anger | > 1 | .0418 |
| Distraction‑Sadness | > 2 | .2220 |
| Reappraisal‑Sadness | > 2 | .2166 |
| Expressive Suppression-Sadness | > 2 | .7115 |
| Support Seeking‑Sadness | > 2 | .2821 |
| Rumination‑Sadness | > 2 | .2229 |
| Control‑Sadness | > 2 | .8635 |
| Dysregulation‑Sadness | > 2 | .7950 |
| Distraction‑Fear | > 2 | .9353 |
| Reappraisal‑Fear | > 2 | .0924 |
| Expressive Suppression‑Fear | > 2 | .7534 |
| Support Seeking‑Fear | > 2 | .6533 |
| Rumination‑Fear | > 2 | .1471 |
| Control‑Fear | > 2 | .2064 |
| Dysregulation‑Fear | > 2 | .8603 |
| Distraction‑Anger | > 2 | .1836 |
| Reappraisal‑Anger | > 2 | .5201 |
| Expressive Suppression‑Anger | > 2 | .3302 |
| Support Seeking‑Anger | > 2 | .4741 |
| Rumination‑Anger | > 2 | .2717 |
| Control‑Anger | > 2 | .6705 |
| Dysregulation‑Anger | > 2 | .6146 |

*Supplementary Table S4*

Descriptive Statistics and internal consistency coefficients of study variables

| **Measure** | **(Sub)scale** | **M** | **SD** | **SK** | **KU** | **Min.** | **Max.** | **α** |
| --- | --- | --- | --- | --- | --- | --- | --- | --- |
| SDQ | Prosociality | 1.460 | 0.413 | -0.676 | -0.082 | 0.000 | 2.000 | .677 |
|  | Externalizing | 0.592 | 0.337 | 0.381 | -0.392 | 0.000 | 1.600 | .673 |
|  | Internalizing | 0.680 | 0.369 | 0.350 | -0.559 | 0.000 | 1.800 | .707 |
| PID5BF | Negative Affectivity | 1.214 | 0.647 | 0.134 | -0.712 | 0.000 | 2.800 | .603 |
|  | Detachment | 1.251 | 0.696 | 0.233 | -0.595 | 0.000 | 3.000 | .653 |
|  | Antagonism | 0.814 | 0.608 | 0.768 | 0.155 | 0.000 | 2.800 | .643 |
|  | Disinhibition | 0.893 | 0.616 | 0.407 | -0.565 | 0.000 | 2.800 | .685 |
|  | Psychoticism | 1.019 | 0.730 | 0.356 | -0.683 | 0.000 | 3.000 | .757 |
| WHODAS-12 | Disability | 1.870 | 0.631 | 0.704 | -0.019 | 1.000 | 4.080 | .832 |
| CERSA-Sadness | Distraction‑Sadness | 3.411 | 1.491 | 0.333 | -0.552 | 1.000 | 7.000 | .838 |
|  | Reappraisal‑Sadness | 4.108 | 1.558 | -0.099 | -0.715 | 1.000 | 7.000 | .805 |
|  | Expressive Suppression-Sadness | 3.908 | 1.891 | 0.062 | -1.166 | 1.000 | 7.000 | .877 |
|  | Support Seeking‑Sadness | 2.733 | 1.577 | 0.705 | -0.369 | 1.000 | 7.000 | .819 |
|  | Rumination‑Sadness | 2.730 | 1.520 | 0.771 | -0.165 | 1.000 | 7.000 | .808 |
|  | Control‑Sadness | 5.115 | 1.333 | -0.493 | -0.305 | 1.000 | 7.000 | .735 |
|  | Dysregulation‑Sadness | 2.323 | 1.208 | 0.978 | 0.610 | 1.000 | 7.000 | .672 |
| CERSA-Fear | Distraction‑Fear | 3.368 | 1.572 | 0.308 | -0.697 | 1.000 | 7.000 | .827 |
|  | Reappraisal‑Fear | 3.918 | 1.614 | 0.049 | -0.731 | 1.000 | 7.000 | .841 |
|  | Expressive Suppression‑Fear | 4.043 | 1.740 | 0.007 | -0.961 | 1.000 | 7.000 | .838 |
|  | Support Seeking‑Fear | 3.591 | 1.774 | 0.153 | -1.051 | 1.000 | 7.000 | .842 |
|  | Rumination‑Fear | 3.483 | 1.657 | 0.209 | -0.966 | 1.000 | 7.000 | .806 |
|  | Control‑Fear | 4.980 | 1.390 | -0.351 | -0.566 | 1.000 | 7.000 | .818 |
|  | Dysregulation‑Fear | 2.372 | 1.236 | 0.799 | -0.018 | 1.000 | 6.250 | .712 |
| CERSA-Anger | Distraction‑Anger | 3.150 | 1.577 | 0.508 | -0.462 | 1.000 | 7.000 | .841 |
|  | Reappraisal‑Anger | 3.148 | 1.632 | 0.481 | -0.588 | 1.000 | 7.000 | .867 |
|  | Expressive Suppression‑Anger | 3.904 | 1.743 | 0.141 | -0.978 | 1.000 | 7.000 | .831 |
|  | Support Seeking‑Anger | 3.039 | 1.728 | 0.509 | -0.788 | 1.000 | 7.000 | .848 |
|  | Rumination‑Anger | 3.752 | 1.595 | 0.082 | -0.818 | 1.000 | 7.000 | .756 |
|  | Control‑Anger | 4.561 | 1.497 | -0.199 | -0.527 | 1.000 | 7.000 | .796 |
|  | Dysregulation‑Anger | 2.917 | 1.557 | 0.624 | -0.385 | 1.000 | 7.000 | .794 |
| *Note.* M = Mean, SD = Standard Deviation, SK = Skewness, KU = Kurtosis. | | | | | | | | |

*Supplementary Table S5*

Full-pipeline bootstrap inference for all emotion regulation components predicting cluster membership.

| **Predictor** | **Threshold** | **Estimate^a^** | **95% Bootstrap CI^b^** | **OR^c^** | **95% OR CI** |
| --- | --- | --- | --- | --- | --- |
| Dysregulation-Anger | logit [P ≤ 1] | –0.400 | **[−0.631, −0.079]** | 0.744 | [0.532, 0.924] |
|  | logit [P ≤ 2] | –0.480 | **[−0.701, −0.229]** | 0.637 | [0.496, 0.795] |
| Dysregulation-Fear | logit [P ≤ 1] | –0.362 | **[−0.610, −0.040]** | 0.782 | [0.544, 0.964] |
|  | logit [P ≤ 2] | –0.231 | [−0.429, 0.000] | 0.825 | [0.651, 1.000] |
| Dysregulation-Sadness | logit [P ≤ 1] | –0.480 | **[−0.654, −0.144]** | 0.720 | [0.520, 0.866] |
|  | logit [P ≤ 2] | –0.298 | [−0.454, 0.000] | 0.798 | [0.635, 1.000] |
| Rumination-Anger | logit [P ≤ 1] | –0.313 | **[−0.519, −0.056]** | 0.751 | [0.595, 0.946] |
|  | logit [P ≤ 2] | –0.217 | [−0.426, 0.000] | 0.862 | [0.653, 1.000] |
| Expressive Suppression-Sadness | logit [P ≤ 1] | –0.095 | [−0.331, 0.014] | 0.909 | [0.718, 1.014] |
|  | logit [P ≤ 2] | –0.286 | [−0.480, 0.000] | 0.772 | [0.619, 1.000] |
| Reappraisal-Anger | logit [P ≤ 1] | 0.080 | [0.000, 0.259] | 1.000 | [1.000, 1.296] |
|  | logit [P ≤ 2] | 0.313 | **[0.070, 0.554]** | 1.350 | [1.073, 1.740] |
| Support Seeking-Sadness | logit [P ≤ 1] | 0.441 | **[0.079, 0.662]** | 1.383 | [1.082, 1.938] |
|  | logit [P ≤ 2] | 0.090 | [0.000, 0.324] | 1.000 | [1.000, 1.383] |
| Rumination-Fear | logit [P ≤ 1] | –0.174 | [−0.383, 0.000] | 0.886 | [0.682, 1.000] |
|  | logit [P ≤ 2] | –0.209 | [−0.433, 0.000] | 0.812 | [0.649, 1.000] |
| Support Seeking-Anger | logit [P ≤ 1] | 0.180 | [0.000, 0.383] | 1.183 | [1.000, 1.467] |
|  | logit [P ≤ 2] | 0.066 | [0.000, 0.289] | 1.000 | [1.000, 1.335] |
| Expressive Suppression-Fear | logit [P ≤ 1] | -0.170 | [-0.372, 0.000] | 0.863 | [0.690, 1.000] |
|  | logit [P ≤ 2] | 0.019 | [-0.167, 0.000] | 1.000 | [0.847, 1.000] |
| Reappraisal-Fear | logit [P ≤ 1] | 0.201 | [0.000, 0.478] | 1.182 | [1.000, 1.613] |
|  | logit [P ≤ 2] | 0.152 | [0.000, 0.447] | 1.150 | [1.000, 1.564] |
| Reappraisal-Sadness | logit [P ≤ 1] | -0.095 | [-0.298, 0.000] | 1.000 | [0.742, 1.000] |
|  | logit [P ≤ 2] | 0.129 | [-0.037, 0.320] | 1.099 | [0.964, 1.377] |
| Support Seeking-Fear | logit [P ≤ 1] | 0.014 | [0.000, 0.244] | 1.000 | [1.000, 1.277] |
|  | logit [P ≤ 2] | 0.214 | [0.000, 0.396] | 1.169 | [1.000, 1.486] |
| Distraction-Sadness | logit [P ≤ 1] | -0.146 | [-0.341, 0.000] | 0.928 | [0.711, 1.000] |
|  | logit [P ≤ 2] | 0.159 | [0.000, 0.383] | 1.146 | [1.000, 1.466] |
| Expressive Suppression-Anger | logit [P ≤ 1] | -0.194 | [-0.420, 0.000] | 0.817 | [0.657, 1.000] |
|  | logit [P ≤ 2] | -0.130 | [-0.300, 0.000] | 1.000 | [0.741, 1.000] |
| Control-Fear | logit [P ≤ 1] | -0.007 | [0.000, 0.213] | 1.000 | [1.000, 1.238] |
|  | logit [P ≤ 2] | 0.125 | [-0.011, 0.323] | 1.072 | [0.989, 1.382] |
| Distraction-Anger | logit [P ≤ 1] | NA | [0.000, 0.417] | 1.000 | [1.000, 1.518] |
|  | logit [P ≤ 2] | NA | [-0.352, 0.000] | 1.000 | [0.703, 1.000] |
| Distraction-Fear | logit [P ≤ 1] | NA | [-0.442, 0.000] | 1.000 | [0.643, 1.000] |
|  | logit [P ≤ 2] | NA | [-0.468, 0.000] | 1.000 | [0.627, 1.000] |
| Control-Sadness | logit [P ≤ 1] | NA | [0.000, 0.244] | 1.000 | [1.000, 1.277] |
|  | logit [P ≤ 2] | NA | [-0.007, 0.174] | 1.000 | [0.993, 1.190] |
| Rumination-Sadness | logit [P ≤ 1] | NA | [-0.155, 0.000] | 1.000 | [0.856, 1.000] |
|  | logit [P ≤ 2] | NA | [-0.219, 0.000] | 1.000 | [0.803, 1.000] |
| Control-Anger | logit [P ≤ 1] | NA | [0.000, 0.263] | 1.000 | [1.000, 1.301] |
|  | logit [P ≤ 2] | NA | [0.000, 0.151] | 1.000 | [1.000, 1.163] |
| *Note.* OR = odds ratio of cluster membership at the given threshold (exp of unconditional bootstrap median). CIs excluding zero are bold-faced.  ^a^Point estimates are unpenalized coefficients from the full-data model, representing the best available effect size estimates given the observed variable selection; because the CIs are derived from a zero-inflated unconditional distribution rather than the full-data estimate, they are asymmetric around the point estimates. This is expected and by design, not an error.  ^b^Bootstrap CIs are unconditional (mass-at-zero for non-selected resamples included); they account for full-pipeline selection uncertainty by incorporating both the probability of a predictor being selected and the uncertainty of its post-selection estimate.  ^c^Because the cumulative logit model predicts the probability of being in a lower severity category, an OR < 1 indicates that higher predictor scores are associated with a higher risk of severe dysfunction, whereas an OR > 1 indicates increased odds of remaining in a lower-dysfunction cluster. | | | | | |

*Supplementary Table S6*

Ordinal LASSO Regression Predicting Two-Cluster Membership and Bootstrapped Selection Frequencies (Sensitivity Analysis).

| **Variables** | **\|β\| (λ_1se_)ᵃ** | **# Zero Thresholdsᵇ** | **Selection frequencyᶜ** |
| --- | --- | --- | --- |
| Dysregulation-Anger | 0.358 | 0 | 1.000 |
| Dysregulation-Fear | 0.325 | 0 | 0.999 |
| Expressive Suppression-Sadness | 0.236 | 0 | 0.996 |
| Dysregulation-Sadness | 0.212 | 0 | 0.981 |
| Reappraisal-Anger | 0.206 | 0 | 0.977 |
| Rumination-Anger | 0.140 | 0 | 0.907 |
| Reappraisal-Fear | 0.129 | 0 | 0.871 |
| Support Seeking-Fear | 0.091 | 0 | 0.743 |
| Rumination-Fear | 0.091 | 0 | 0.778 |
| Support Seeking-Sadness | 0.070 | 0 | 0.732 |
| Rumination-Sadness | 0.024 | 0 | 0.575 |
| Reappraisal-Sadness | 0.019 | 0 | 0.513 |
| Support Seeking-Anger | 0.014 | 0 | 0.516 |
| Distraction-Sadness | 0.000 | 1 | 0.100 |
| Control-Sadness | 0.000 | 1 | 0.234 |
| Distraction-Fear | 0.000 | 1 | 0.163 |
| Expressive Suppression-Fear | 0.000 | 1 | 0.123 |
| Control-Fear | 0.000 | 1 | 0.465 |
| Distraction-Anger | 0.000 | 1 | 0.020 |
| Expressive Suppression-Anger | 0.000 | 1 | 0.418 |
| Control-Anger | 0.000 | 1 | 0.039 |
| *Note.* ^a^Absolute value of the LASSO coefficient at λ_1se_, reported for ranking purposes only. These values are subject to shrinkage bias and should not be interpreted as unbiased effect-size estimates; formal statistical inference is conducted on the full-pipeline bootstrap.  ^b^Number of thresholds (out of one) for which the coefficient was shrunk exactly to zero at λ1se.  ^c^Proportion of full-pipeline resamples (B = 10000) in which the predictor received a nonzero LASSO coefficient. Predictors with selection frequency > 9500 are classified as stable and carried forward to bootstrap inference (Table S7). | | | |

*Supplementary Table S7*

Full-Pipeline Bootstrap Inference for Stable Emotion Regulation Predictors of Two-Cluster Membership (B = 10000, Sensitivity Analysis).

| **Predictor** | **Estimate** | **95% Bootstrap CI^a^** | **OR^b^** | **95% OR CI** |
| --- | --- | --- | --- | --- |
| Dysregulation-Anger | –0.438 | **[–0.713, –0.212]** | 0.629 | [0.490, 0.809] |
| Dysregulation-Fear | –0.410 | **[–0.660, –0.141]** | 0.672 | [0.517, 0.868] |
| Dysregulation-Sadness | –0.316 | **[–0.552, –0.075]** | 0.733 | [0.576, 0.927] |
| Expressive Suppression-Sadness | –0.375 | **[–0.587, –0.099]** | 0.710 | [0.556, 0.906] |
| Reappraisal-Anger | 0.280 | **[0.039, 0.578]** | 1.371 | [1.040, 1.782] |
| *Note.* OR = odds ratio of cluster membership (high vs. low dysfunction; exp of unconditional bootstrap median). CIs excluding zero are bold-faced.  ^a^Bootstrap CIs are unconditional (mass-at-zero for non-selected resamples included), accounting for full-pipeline selection uncertainty.  ^b^Because the cumulative logit model predicts the probability of being in a lower severity category, an OR < 1 indicates that higher predictor scores are associated with a higher risk of severe dysfunction, whereas an OR > 1 indicates increased odds of remaining in a lower-dysfunction cluster. | | | | |


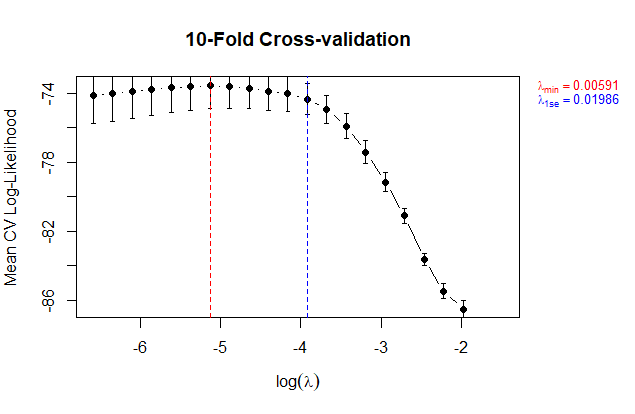


**Supplementary Figure S1.** Cross-validation plot.


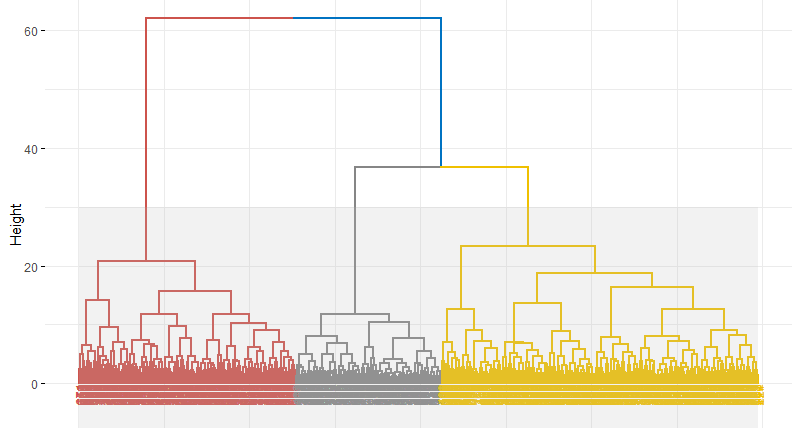


**Supplementary Figure S2.** Dendrogram plot for hierarchical clustering using Ward’s method with squared Euclidean distances.


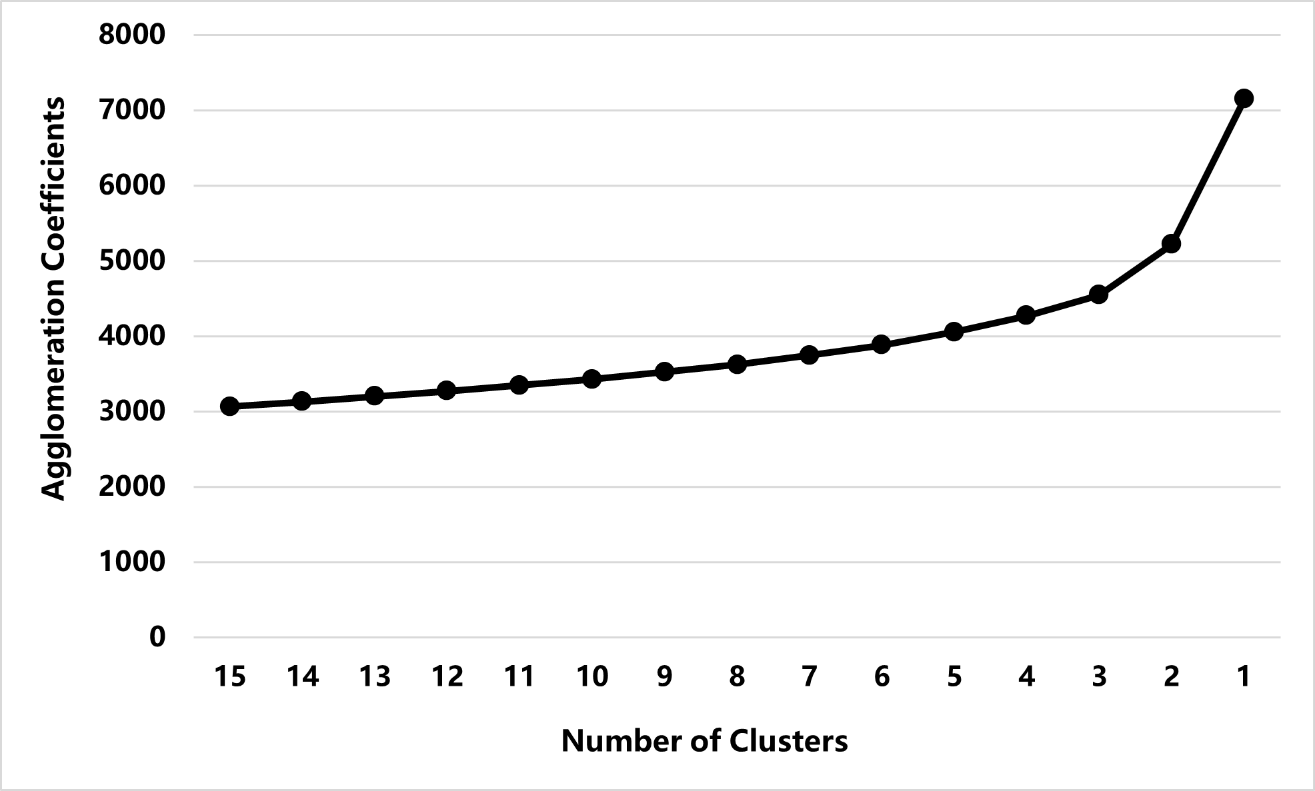


**Supplementary Figure S3.** Agglomeration schedule plot (last 15 steps).
